# Synergistic Effects in the Gas Sensitivity of Polypyrrole/Single Wall Carbon Nanotube Composites

**DOI:** 10.3390/s120607965

**Published:** 2012-06-08

**Authors:** Duong Ngoc Huyen, Nguyen Trong Tung, Tran Dinh Vinh, Nguyen Duc Thien

**Affiliations:** School of Engineering Physics, Hanoi University of Science and Technology, No.1, Daicoviet, Hanoi 10000, Vietnam; E-Mails: trongtung227@yahoo.com (N.T.T.); trandinhvinhht@yahoo.com (T.D.V.); ducthien_ly@yahoo.com (N.D.T.)

**Keywords:** PPy, single wall carbon nanotube, nanocomposite, gas sensor

## Abstract

Polypyrrole/single wall carbon nanotube composites were synthesized by *in-situ* chemical polymerization using pyrrole (PPy) as precursor and single wall carbon nanotubes (SWNTs) as additive component. Electron microscope images reveal that SWNTs component acts as nucleation sites for PPy growth in the form of spherical and cylindrical core-shell structures. The SWNTs/PPy core-shell results in thin *n-p* junctions which modify the PPy bandgap and reduce the work function of electrons. As a result of the strong coupling, Raman and IR spectra show that the PPy undergoes a transition from polaron to bipolaron state, *i.e.*, indicating an increase in the conductivity. In the UV-Vis spectra, the 340 nm adsorption band (π*-π transition) exhibits a red shift, while the 460 nm adsorption band (bipolaron transition) experiences a blue shift indicating a change in electronic structure and a relocation of polaron levels in the band gap of PPy. The modification in PPy electronic structure brings in a synergistic effect in sensing feature. Upon exposure to oxygen (an oxidizing agent) and NH_3_ gas (a reducing agent), the PPy/SWNTs nanocomposite shows an enhancement in sensitivity exceeding ten folds in comparison with those of PPy or SWNTs.

## 1. Introduction

The conducting features of intrinsically conductive polymers (ICPs) have led to intensive experimental and theoretical studies worldwide with the expectation of developing many potential applications in rechargeable batteries, microelectronics, light-emitting, photovoltaic devices, sensors, *etc.* [1]. Because the linear π-conjugated systems of ICPs are highly susceptible to chemical or physical doping the electrical properties of the ICPs can be tuned and modified by controlling the this doping. This characteristic also enables the ICPs to be excellent sensing materials for the development of a variety of gas sensors [2–4]. Among the ICPs, polypyrrole (PPy) and its derivatives have attracted a great deal of attention because of its high electrical conductivity, good environmental stability and simple synthesis and processing. Furthermore, PPy is among a small number of materials demonstrating gas sensing features at room temperature, a fascinating prospect for developing practical applications. In order to further improve the sensing performance and facilitate the processing, the coupling of PPy with variety of inorganic and organic materials in a composite has been widely used as an effective approach [5,6]. The combination of component properties has been observed in the resulting composites with some synergistic effects. For example, the combination of PPy with oxides (Fe_2_O_3_, SnO_2_, TiO_2_, WO_3_, *etc.*), polymers, and nanostructures has produced hybrid composite materials with higher sensitivity, wider selectivity and lower working temperature [7–13]. Continuous studies are being carried on to gain more insights into the PPy based composites and to realize effective gas sensor devices with better and better performance.

Carbon nanotubes (CNTs) [14,15], new allotropic forms of carbon with a unique morphology involving an all surface reacting structure offer great potential for gas sensing devices. However, the lack of selectivity, long recovery and less than expected ideal sensitivity have limited the practical application of CNTs. Hybrids of CNTs with suitable components also have been proposed as active approaches to improve CNTs performance. With the same π-conjugated systems dominating the resulting structures, the strong coupling of CNTs and ICPs via noncovalent or π-π interactions is expected to produce new nanocomposites with excellent gas sensing features [16–18]. In particular, the combination of PPy and SWNTs in nanocomposites has shown significant improvements in the sensing performance of PPy [19–22]. The impact is considered to arise from the interfacial interaction between PPy and SWNTs, however the nature and the mechanism of the interactions (including the environmental contact surface) are quite complex and not yet fully understood. In this work, an effort was made to further study the effect of SWNTs additive on the properties of PPy in PPy/SWNTs nanocomposites that were synthesized by *in situ* chemical polymerization.

## 2. Experimental Procedure

Pyrrole (Py) 99.5% (Aldrich. Co.), ammonium persulfat (APS, Kanto Chemical Co. Inc.) and AP-grade SWNTs (ILJIL Co. Korea) were used in the experiment. The nanocomposite was synthesized in two steps as follows: firstly, SWNTs (20.0 mg) were chemically purified in HCl (5.0 mL, 37% Aldrich) at 120 °C for 3 h and then 3 h in an ultrasonic bath at room temperature. After filtering and extraction from the acidic solution, the remain SWNTs (around 5.0 mg) then were dispersed into isopropanol/distilled water solution of 1.0 M HCl and 0.1 M Py (30.0 mL) in an ultrasonic bath at 0 °C. Secondly, the polymerization was carried out at 0 °C by mixing equivolumetric solutions of 0.1 M APS and 1.0 M HCl. After 1.5 h of continuously agitating by a magnetic stirrer the polymerization was terminated by pouring ethanol into the mixture. The resulting black nanocomposites formed in the solution were filtered, cleaned with distilled water, rinsed with and kept in 0.1 M HCl solution.

The morphology of the nanocomposite was characterized by a FESEM (Hitachi S4800) and TEM (Jeol TEM). FTIR and Raman spectra were recorded on a Nicolet 6700 NRX FT-Raman Module Spectrometer. The FTIR spectra were measured in the range 400–4,000 cm^−1^ at a resolution of 2 cm^−1^ using pressed KBr pellets. The Raman spectra were carried out with a 1064 nm excitation laser at a resolution of 4 cm^−1^. The test sensing sample was fabricated by drop coating of a solution of PPy or PPy/SWNTs nanocomposite (10 mg/mL) onto Pt interdigitated electrodes which were screen printed on alumina substrate. The test sample of 110–200 nm in thickness then was dried at 70–80 °C in open air to evaporate the residual solvents. The sensitivity was determined by the percentage change in materials resistance ΔR/R_ini_, where ΔR = R − R_ini_, R_ini_ is initial sample resistance while R is sample resistance upon exposure to the target gas. The test sample was put in a closed chamber with installed electrodes, gas inlet and outlet. The target gases were oxygen (oxidizing agent) and ammonia (NH_3_) (reducing agent) in the environment at room temperature. The experimental data were acquired and stored by a computer with a data acquisition board (DAQ) and the assistance of Data Studio software.

## 3. Results and Discussion

**Figure 1.**
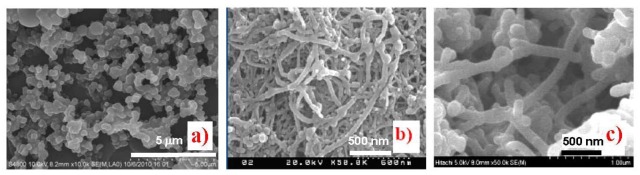
FESEM images of (**a**) PPy, scale bar 1 μm; (**b**) SWNTs, scale bar 500 nm; (**c**) PPy/SWNTs nanocomposite, scale bar 500 nm.

**Figure 2.**
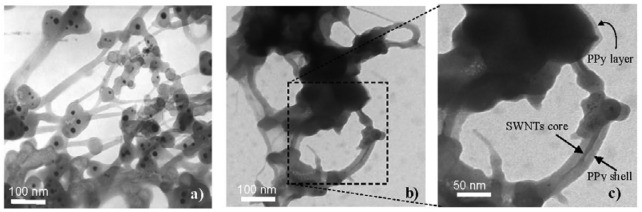
TEM images of SWNTs (**a**); PPy/SWNTs nanocomposites (**b**) and PPy layer/shell on the surface of amorphous carbon and out of SWNTs bundle core (**c**).

Experiments show that the presence of additive SWNTs causes a significant change in the PPy morphology. As can be seen from SEM and TEM images (Figures 1 and 2), the pure PPy has a granular structure with a mean size of around 400–500 nm, but in the nanocomposite the resulting PPy coats the surface of SWNTs structures with PPy thin layers resulting in spherical and cylindrical forms. From the chemical point of view, the radical monomers and oligomers at the first oxidization stage are more active preferably adsorbed and anchored at strong binding sites on the SWNTs and amorphous carbon surfaces that act as nucleation sites. In the following stage, PPy chains grow on the surface of SWNTs and amorphous carbon forming core-shell structures (Figure 2(b,c)). The spherical structures are believed to arise from amorphous carbon cores while the cylindrical forms have nanotube cores. The thickness of the PPy shell is estimated around 15–20 nm. Carbon nanotubes in general are good electron acceptors while PPy can be considered as a donor the forming PPy/SWNTs core-shell could be resulted from the charge transfer complex. The interfacial adhesion of PPy on surface of SWNTs shows evidence of strong bonds between carbon nanotubes and radical Py monomers via donor-acceptor interaction [16,17]. Depending on the synthesis condition, the preferable anchoring sites changes resulting in the different morphologies, as shown in Figure 1(c). The interfacial interaction between SWNTs, amorphous carbon and PPy in the nanocomposites is expected to modify their chemical and electronic structures.

**Figure 3.**
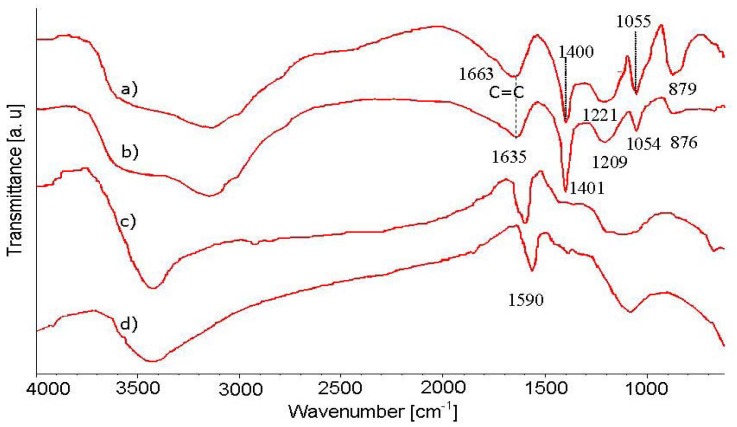
FTIR spectra images of SWNTs and PPy/SWNTs nanocomposites: (**a**) PPy; (**b**) PPy /SWNTs nanocomposite; (**c**) AP-grade SWNTs and (**d**) purified SWNTs.

The FTIR spectra of PPy, PPy/SWNTs AP-grade SWNTs, and purified SWNTs are shown in Figure 3. All of the spectra contain a broad adsorption band in the domain between 4,000 and 2,500 cm^−1^, which is commonly assigned for the adsorption band of O-H, C-H, N-H groups. The adsorption band around 1,590 cm^−1^ in the SWNTs spectrum represents the C=C bond vibration on the SWNTs walls. The 1,663 and 1,635 cm^−1^ bands, respectively, present in PPy and PPy/SWNTs spectra likely represent the C=C bond and/or C=N bond which represent the oxidizing state of PPy. The sharp adsorption band around 1,400 cm^−1^ corresponds to the in-plane deformation of the N-H bond. The 1,206 and 917 cm^−1^ adsorption bands are assigned for the ring deformation and ring breathing, respectively. The 1,050 and 880 cm^−1^ adsorption bands correspond to the in-plane deformation and out-of plane vibration of the C-H bond, respectively. The 1,560 and 1,480 cm^−1^ adsorption bands which are assigned for stretching vibrations and ring breathing of C=C/C−C are hardly observed in the spectra which may be due to the over oxidization of PPy in the HCl environment. As can be seen from the FTIR spectra in Figure 3(a,b), the specific peaks representing PPy adsorption bands in the PPy/SWNTs spectrum undergo a red shift to longer wavelength and experience a change in intensities, indicating an interaction between PPy and SWNTs. The double bonds (C=C and C=N) and the ring deformation showing a larger red shift in the adsorption band (according to the wavenumber drift from 1,663 to 1,635 cm^−1^ and from 1,221 to 1,209 cm^−1^, respectively) are likely the most affected PPy sites in the nanocomposite.

**Figure 4.**
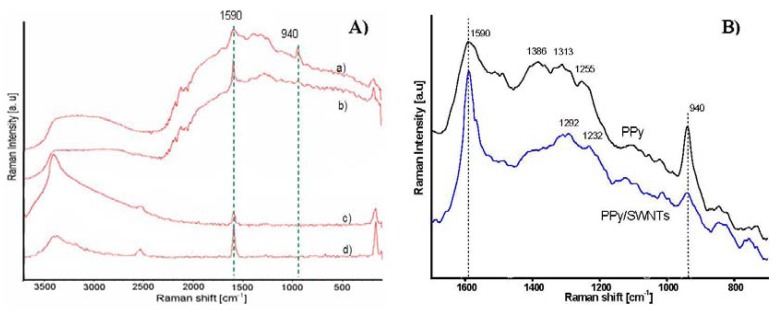
(**A**) Raman spectra of PPy (a), PPy/SWNTs (b), SWNTs (c), purified SWNTs (d) at 1,064 nm laser excitation; (**B**) Raman spectra of PPy and PPy/SWNTs in fingerprint spectral region.

The Raman spectra of PPy PPy/SWNTs, AP-grade SWNTs, and purified SWNTs, are shown in Figure 4. The striking points in the spectra are the appearance of a 940 cm^−1^ peak in the PPy spectrum and the increases in the 1,590 and 1,290 cm^−1^ peaks in the PPy/SWNTs spectrum. From the Raman assignment of PPy, the band located at about 940 cm^−1^ is assigned to the ring deformation associated with radical cation (polaron state). The band around 1,590 cm^−1^ is likely the superposition of tangential mode (G-band) of the carbon atoms of SWNTs and the G-band associated with sp^2^-hybridized carbon atoms (C=C bond) of PPy. The bands at approximately 1,310, 1,250 and 1,380 cm^−1^ are attributed to the ring-stretching mode, which are assigned to the oxidized species of PPy. The 1,290 cm^−1^ band in PPy/SWNTs is assumed to be the superposition of both 1,310 and 1,253 cm^−1^ band which stand for the bipolaron state of PPy [15]. The decline of 940 cm^−1^ band and the emersion of 1,290 cm^−1^ band in Raman spectra then indicate the transition from polaron to bipolaron state, *i.e.*, indicate an increase in conductivity of PPy.

The broad band around 3,000–3,700 cm^−1^ is also observed in the Raman spectra. Due to the fact that the band intensity relatively increases with the laser excitation intensity, other than the assignment of vibration modes of O-H, N-H and C-H groups, a portion of the 3,000–3,700 cm^−1^ band is speculated to belong to the infrared active vibrational modes [23]. Resulting from the self-localization of polaronic and bipolaronic states, the broadening of band width towards longer wavelengths implies that the SWNTs-PPy interaction brings more new polaron and bipolaron levels into the band gap of SWNTs/PPy nanocomposites.

**Figure 5.**
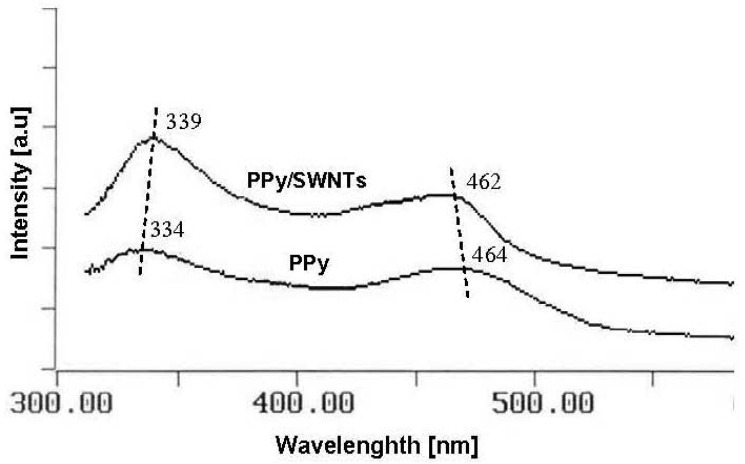
UV-Vis spectra of PPy and PPy/SWNTs nanocomposite.

Figure 5 shows the UV-Vis spectra of PPy and PPy/SWNTs, in which both spectra contain two broad bands centered around 340 nm and 460 nm. The 340 nm adsorption band represents the π*-π transition and the 460 nm adsorption band corresponds to the bipolaronic transition which are the feature of the oxidized state of PPy segments. As can be seen from the spectra, the π*-π band is intensified and undergoes a red shift from 334 to 339 nm while the bipolaronic transition band exhibits a slight blue shift from 464 to 462 nm when the SWNTs additive component is added. The change in the intensity and position of these adsorption bands indicates a modification in electronic structure and a relocation of polaron levels in PPy band gap due to the interaction with SWNTs.

**Figure 6.**
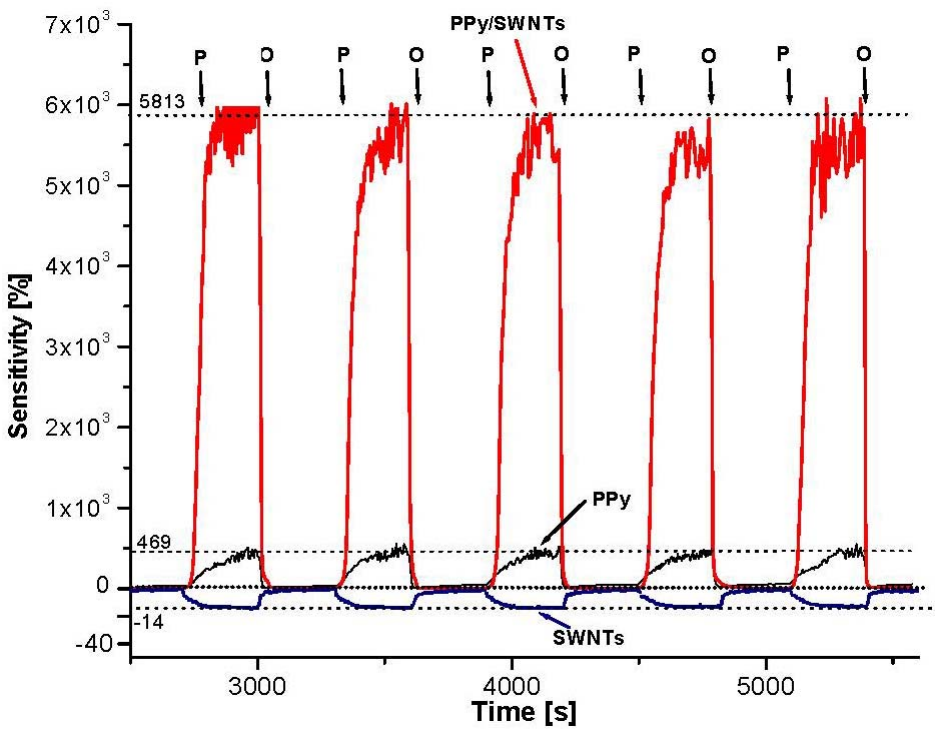
Oxygen sensitivity of PPy, SWNTs and PPy/SWNTs upon exposure to open air (P: low air presure as pump on, O: open air).

It is well known that the conductivity of both components (PPy, SWNTs) and resulting nanocomposites are sensitive to oxygen (oxidizing agent) and NH_3_ (reducing agent). Changing the air pressure as a means to change the oxygen concentration (21% by volume) in the environment, the conductivity of the materials is varied. The O_2_ sensing profiles of PPy, PPy/SWNTs in Figure 6 show the sample resistance rapidly increasing as air pressure reduces to 5.10^2^ Pa (the vacuum pump on) and fast returning back to the origin value as air pressure restored to 10^5^ Pa (the vacuum pump off). The decrease in air pressure will reduce the adsorbed oxygen in the sample surface and *vice versa*. The sample resistance increases as a result of adsorbed oxygen being degassed. The O_2_ sensitivity is also calculated as ΔR/R_ini_, where ΔR = R − R_ini_, R_ini_ is initial sample resistance in open air, while R is the sample resistance at low air pressure. As can be seen from plot, the O_2_ sensitivity of the PPy/SWNTs is roughly 60-fold (6,000%) while the O_2_ sensitivity of the PPy is about 5-fold (500%). However, the SWNTs show a negative response mode with much lesser sensitivity (−14%). Typically, O_2_ sensitivity of PPy/SWNTs nanocomposites is 10–15 times higher than those of PPy. The response time (during degassing) of the PPy/SWNTs is faster than that of PPy (76 and 144 s respectively) while the recovery time (open to the air) of both samples is the same, around 13 s. From the physical point of view, the change in conductivity involves the extraction of electrons from semiconducting PPy and SWNTs as a result of oxygen interaction (physical adsorption) thus converting the materials into p-type semiconductors. The different sensitivity indicates that the majority of charge carriers in SWNTs are likely electrons, *i.e.*, SWNTs are *n*-type semiconductors. The adsorption and desorption of oxygen molecules on the material surface then causes the variation in its conductivity. The more the oxygen concentration in the environment, the more electrons are extracted, and as a result, the material conductivity is increased and *vice versa*. The fast response, recover and the baseline stability indicate that physical adsorptions with weak bonding are dominant in the oxygen-nanocomposites interaction. However, due to the core-shell structure of the nanocomposites, the PPy shell likely take the most share in the sensitivity. In this core-shell structure when SWNTs (conductor or semiconductor) are brought into contact with PPy semiconductor, a space charge region is created. The diffusion of electrons from the SWNTs (either conductors with low work function of 0.2 to 0.6 eV, or semiconductors with narrow band gap of ∼0.5 eV) [24] into PPy region (high work function ∼5 eV and moderate band gap ∼3.1 eV) [3,25] will strongly affect the PPy band gap along the 15–20 nm width. Since the depletion region width is expected to be larger [3], the work function of electrons on the surface of PPy shell then will be reduced and easier to be donated. Higher oxygen sensitivity indicates the fact that more oxygen adsorption sites are created on the PPy shell surface.

**Figure 7.**
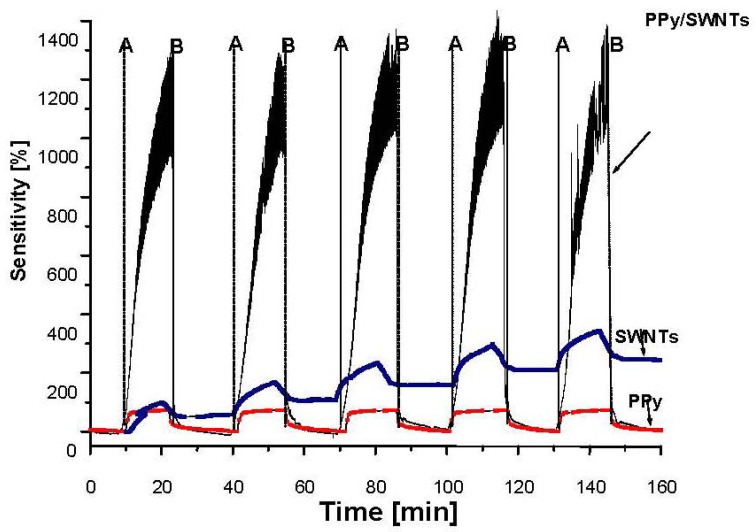
NH_3_ sensitivity of PPy, SWNTs and PPy/SWNTs upon exposure to 750 ppm NH_3_. (A: NH_3_ on, B: NH_3_ gas off).

On the other hand, upon exposure to a reducing agent such as NH_3_, the conductivity of the materials is decreased. The NH_3_ interaction is equivalent to an injection of electrons into the valence band of *p*-type semiconducting SWNTs and PPy and this reduces the conductivity of the materials. As can be seen from Figure 7, the NH_3_ sensitivity of PPy/SWNTs nanocomposite upon exposure to 750 ppm of NH_3_ is about 1000% higher, ten-fold more than those of PPy (110%) and SWNTs (80%). However, PPy and SWNTs show faster response than PPy/SWNTs. The response times of PPy, SWNTs and PPy/SWNTs are 15, 40 and 415 s while the recovery times of PPy, SWNTs and PPy/SWNTs are 8, 20 and 20 s, respectively. From the chemical viewpoint, the gas sensitivity relates to the number of gas adsorbed molecules, in turn proportional to the adsorption sites on the material surface. For the case of PPy, SWNT and PPy/SWNTs gas sensing samples, the conductivity relies on electron acceptors or donors, that is it relies on the nature of adsorbed gases (oxidizing or reducing). Upon exposure to the open air all the materials are converted into *p*-type semiconductors due to adsorbed oxygen molecules, electron acceptors. The presence of injected NH_3_ molecules (reducing agent) in the air drives a secondary doping of electron donors as a result of the adsorption of NH_3_ molecules. The high sensitivity to both O_2_ and NH_3_ with inverse nature observed in PPy and PPy/SWNTs systems indicates that the adsorbed O_2_ molecules could be a reason to promote the adsorption of NH_3_ [26]. The higher NH_3_ sensitivity in PPy/SWNTs nanocomposites implies that more NH_3_ adsorption sites (shallow donor and acceptor levels) are created on the PPy surfaces due to the interaction between PPy and SWNTs and adsorbed O_2_ molecules.

## 4. Conclusions

In PPy/SWNTs nanocomposites, the coupling between PPy and SWNTs causes a modification of the PPy structures. The SWNTs surface plays the role of nucleation sites for PPy attached to and growth on forming spherical and cylindrical core-shell structure. The SWNTs/PPy core-shell results in thin *n-p* junctions which modify the PPy band gap and reduce the electron work function. The interfacial PPy-SWNTs interactions promote the polaron to bipolaron transition and the formation of various shallow levels in the band gap of oxidizing PPy. The modification in PPy electronic structure accounts for synergistic effect in its gas sensing feature. The sensitivity of PPy/SWNTs nanocomposite upon exposure to oxygen and ammonium hydrogen gases is ten-fold greater in comparison to those of PPy and SWNTs independently.

## References

[1] Skotheim T.A., Reynolds J.R. (2007). Handbook of Conducting Polymer.

[2] McQuade D.T., Pullen A.E., Swager T.M. (2000). Conjugated polymer-based chemical sensors. Chem. Rev..

[3] Jiri J., Mira J. (2003). Conducting polymers in electronic chemical sensors. Nat. Mater..

[4] Bai H., Shi G.Q. (2007). Review: Gas sensors based on conducting polymers. Sensors.

[5] Karin P.K. (2002). Chemical gas sensors based on organic semiconductor polypyrrole. Crit. Rev. Anal. Chem..

[6] Ram M.K., Bhethanabotla V.R. (2010). Sensors for Chemical and Biological Applications.

[7] Tandon R.P., Tripathy M.R., Arora A.K., Hotchandani S. (2006). Gas and humidity response of iron oxide-Polypyrrole nanocomposites. Sens. Actuators B.

[8] Ram M.K., Yavuz O., Aldissi M. (2005). NO_2_ gas sensing based on ordered ultrathin films of conducting polymer and its nanocomposite. Synth. Met..

[9] Geng L. (2010). Gas sensitivity study of polypyrrole/WO_3_ hybrid materials to H_2_S. Synth. Met..

[10] Nguyen van C., Potje-Kamloth K. (2001). Electrical and NO*_x_* gas sensing properties of metallo-phthalo-cyanine-doped polypyrrole/silicon heterojunctions. Thin Solid Films.

[11] Ihm D.-W., Woo H.-Y., Hwang C.-R., Lee Y.-K., Kim J.-Y. (2011). Fabrication of polypyrrole-phenylalanine nano-films with NH_3_ gas sensitivity. Sens. Actuators B.

[12] Dai L., Soundarrajan P., Kim T. (2002). Sensors and sensor arrays based on conjugated polymers and carbon nanotubes. Pure Appl. Chem..

[13] Mylvaganam K., Zhang L.C. (2007). Fabrication and application of polymer composites comprising carbon nanotubes. Recent Pat. Nanotechnol..

[14] Kroto H.W., Heath J.R., O'Brien S.C., Curl R.F., Smalley R.E. (1985). C60: Buckminsterfullerene. Nature.

[15] Ijima S. (1991). Helical microtubules of graphitic carbon. Nature.

[16] Chen R.J., Zhang Y., Wang D., Dai H. (2001). Noncovalent sidewall functionalization of single-walled carbon nanotubes for protein immobilization. J. Am. Chem. Soc..

[17] Hoeben F.J.M., Jonkheijm P., Meijer E.W., Schenning A.P.H.J. (2005). About supramolecular assemblies of *π*-conjugated systems. Chem. Rev..

[18] Wang Y., Yeow J.T.W. (2009). Review article: A review of carbon nanotubes-based gas sensors. J. Sens..

[19] Chen Y., Li Y., Wang H., Yang M. (2007). Gas sensitivity of a composite of multiwalled carbon nanotubes and polypyrrole prepared by vapor phase polymerization. Carbon.

[20] An K.H., Jeong S.Y., Hwang H.R., Lee Y.H. (2004). Enhanced sensitivity of a gas sensor incorporating single-walled carbon nanocomposites. Adv. Mater..

[21] Hieu N.V., Dung N.Q., Tam P.D., Trung T., Chien N.D. (2009). Thin film polypyrrole/SWCNTs nanocomposites based NH_3_ sensor operated at room temperature. Sens. Actuators B.

[22] Wang Z.M., Tang X.C., Xiao Y.H., Yu X.J., Zhang L., Jia D.Z., Chen G.C. (2011). Polypyrrole coated carbon nanotubes: Preparation, characterization, and gas-sensing properties. J. Inorg. Mater..

[23] Santos M.J.L., Brolo A.G., Girotto E.M. (2007). Study of polaron and bipolaron states in polypyrrole by *in situ* Raman spectroelectrochemistry. Electrochim. Acta.

[24] Suzuki S., Watanabe Y., Homma Y., Fukuba S., Heun S., Locatelli A. (2004). Work functions of individual single-walled carbon nanotubes. Appl. Phys. Lett..

[25] Inganäs O., Skotheim T., Lundström I. (1983). Polypyrrole‐semiconductor Schottky barriers. J. Appl. Phys..

[26] Feng X., Irle S., Witek H., Morokuma K., Vidic R., Borguet E. (2005). Sensitivity of ammonia interaction with single-walled carbon nanotube bundles to the presence of defect sites and functionalities. J. Am. Chem. Soc..

